# Bis(2-amino-6-methyl­pyridinium) *trans*-diaqua­bis­(pyrazine-2,3-dicarboxyl­ato)cobaltate(II) octa­hydrate

**DOI:** 10.1107/S1600536811001127

**Published:** 2011-01-29

**Authors:** Hossein Eshtiagh-Hosseini, Nafiseh Alfi, Masoud Mirzaei, Philip E. Fanwick

**Affiliations:** a Department of Chemistry, School of Sciences, Ferdowsi University of Mashhad, Mashhad, Iran; b Department of Chemistry, Purdue University, W. Lafayette, IN 47907, USA

## Abstract

The title compound, (C_6_H_9_N_2_)_2_[Co(C_6_H_2_N_2_O_4_)_2_(H_2_O)_2_]·8H_2_O, was obtained by the reaction of CoCl_2_·6H_2_O with 1,4-pyrazine-2,3-dicarb­oxy­lic acid and 2-amino-6-methyl­pyridine in aqueous solution (molar ratio 1:2:2). The Co^II^ ion is situated on an inversion centre and is coordinated by two O and two N atoms of two symmetry-related 1,4-pyrazine-2,3-dicarboxyl­ate ligands and two water mol­ecules and has a disorted octa­hedral coordination environment. The asymmetric unit also contains four water mol­ecules. In the crystal, extensive inter­molecular classical N—H⋯O, O—H⋯O and O—H⋯N hydrogen bonds and π–π stacking inter­actions [centroid–centroid distance = 3.490 (1) Å] connect the various components, forming a three-dimensional network.

## Related literature

For related structures based on 1,4-pyrazine-2,3-dicarboxylate ligands, see: Eshtiagh-Hosseini, Alfi *et al.* (2010 ▶). Eshtiagh-Hosseini, Gschwind *et al.* (2010 ▶). Eshtiagh-Hosseini, Necas *et al.* (2010 ▶).
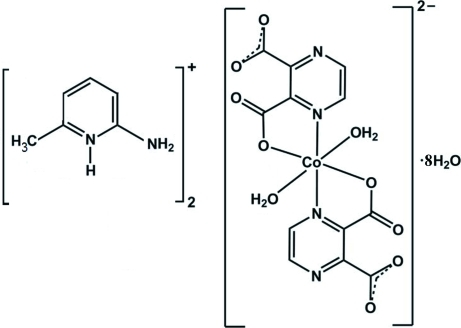


## Experimental

### 

#### Crystal data

(C_6_H_9_N_2_)_2_[Co(C_6_H_2_N_2_O_4_)_2_(H_2_O)_2_]·8H_2_O
*M* *_r_* = 789.58
Triclinic, 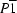

*a* = 6.8570 (4) Å
*b* = 10.2348 (5) Å
*c* = 13.6403 (10) Å
α = 109.604 (4)°
β = 90.424 (5)°
γ = 105.524 (4)°
*V* = 863.89 (9) Å^3^
*Z* = 1
Cu *K*α radiation
μ = 4.68 mm^−1^
*T* = 150 K
0.20 × 0.18 × 0.14 mm

#### Data collection

Rigaku RAPID II diffractometer
Absorption correction: multi-scan (*SCALEPACK*; Otwinowski & Minor, 1997 ▶) *T*_min_ = 0.280, *T*_max_ = 0.508
19137 measured reflections
3152 independent reflections
3151 reflections with > 2.0σ(*I*)
*R*_int_ = 0.036

#### Refinement

*R*[*F* ^2^ > 2σ(*F* ^2^)] = 0.030
*wR*(*F* ^2^) = 0.087
*S* = 1.04
3152 reflections
285 parameters
H atoms treated by a mixture of independent and constrained refinement
Δρ_max_ = 0.23 e Å^−3^
Δρ_min_ = −0.40 e Å^−3^

### 

Data collection: *CrystalClear* (Molecular Structure Corporation & Rigaku, 2001 ▶); cell refinement: *DENZO*/*SCALEPACK* (Otwinowski & Minor, 1997 ▶); data reduction: *DENZO*/*SCALEPACK*; program(s) used to solve structure: *SIR2004* (Burla *et al.*, 2005 ▶); program(s) used to refine structure: *SHELXL97* (Sheldrick, 2008 ▶); molecular graphics: *ORTEP* (Johnson, 1976 ▶) and *PLATON* (Spek, 2009 ▶); software used to prepare material for publication: *SHELXL97* and local programs.

## Supplementary Material

Crystal structure: contains datablocks global, I. DOI: 10.1107/S1600536811001127/bt5444sup1.cif

Structure factors: contains datablocks I. DOI: 10.1107/S1600536811001127/bt5444Isup2.hkl

Additional supplementary materials:  crystallographic information; 3D view; checkCIF report

## Figures and Tables

**Table 1:** Hydrogen-bond geometry (Å, °)

*D*—H⋯*A*	*D*—H	H⋯*A*	*D*⋯*A*	*D*—H⋯*A*
N11—H11⋯O31	0.82 (2)	1.98 (2)	2.794 (3)	179 (2)
N12—H121⋯O2W^i^	0.86 (3)	2.05 (2)	2.900 (2)	170 (2)
N12—H122⋯O32	0.84 (3)	1.97 (3)	2.804 (3)	176 (2)
O1W—H1W1⋯O2W^ii^	0.78 (3)	1.99 (3)	2.770 (3)	178 (3)
O1W—H1W2⋯O5W^ii^	0.85 (3)	1.85 (3)	2.697 (2)	172 (3)
O2W—H2W1⋯O21	0.85 (3)	2.10 (3)	2.942 (3)	168 (3)
O2W—H2W2⋯O4W	0.72 (3)	2.07 (3)	2.784 (3)	176 (2)
O3W—H3W2⋯O4W	0.83 (3)	1.99 (3)	2.811 (3)	167 (3)
O4W—H4W1⋯O3W^iii^	0.80 (3)	1.98 (2)	2.755 (2)	165 (3)
O4W—H4W2⋯O31^iv^	0.80 (2)	1.94 (2)	2.738 (2)	178 (3)
O5W—H5W1⋯O32^v^	0.79 (2)	2.00 (3)	2.767 (2)	161 (3)
O5W—H5W2⋯N4^vi^	0.77 (3)	2.11 (3)	2.871 (3)	167 (3)

Symmetry codes: (i) 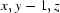; (ii) 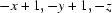; (iii) 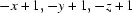; (iv) 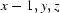; (v) 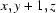; (vi) 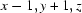.

**Fig. 1.**
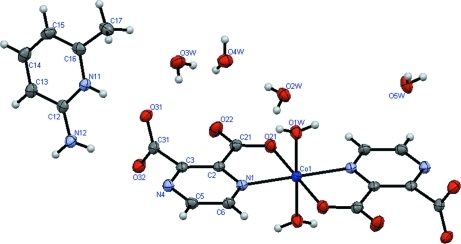
An ORTEP drawing of the title compound showing 50% ellipsoid probability. Only the symmetry independent atoms are labelled.

**Fig. 2.**
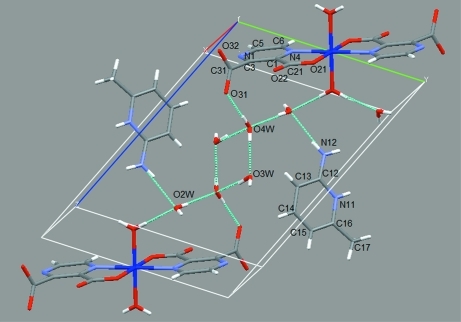
Water molecules connecting anions and cations.

**Fig. 3.**
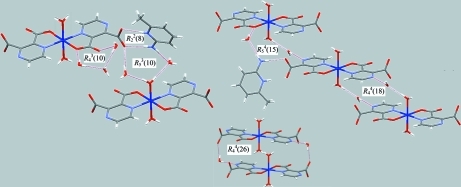
Schematic representation of different graph set motifs in the crystalline network of 1. Dashed lines indicate the hydrogen bonds.

**Fig. 4.**
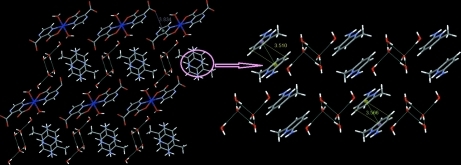
Perspective views of the π–π stacking interactions.

## supplementary crystallographic information

### Comment

In the recent years, our research group has been interested in the synthesis of proton transfer compounds and study of their behavior with metal ions. We have focused on the proton delivery from polycarboxylic acids, which are considered as very good donors and amines as acceptors. Among polycarboxylic acids, 1,4-pyrazine-2,3-dicarboxylic acid (pyzdcH_2_) as a very important carboxylate derivative has attracted much interest in coordination chemistry and it is the one that we utilized widely in our studies (Eshtiagh-Hosseini, Alfi *et al.*, 2010). In order to develop novel systems, we wish to report the first complex of Co^II^ ion with pydcH_2_ as proton donor and 2a-6 m as proton acceptor. PyzdcH_2_ has proved to be well suited for the construction of multidimensional frameworks due to the presence of two adjacent carboxylate groups (O donor atoms) as substituents on the N-heterocyclic pyrazine ring (N donor atoms).

The asymmetric unit of the title compound (Fig. 1), contains half a [Co(pyzdc)_2_(H_2_O)_2_]^2-^ anion, a (2a-6mpyH)^+^ cation, and four uncoordinated water molecules. In the anions, Co^II^ ion has a N_2_O_4_ donor set bond with normal distances and angles. The title compound can be compared with the mono-nuclear coordination compound of Co^II^ ion which has recently been synthesized and characterized by our research group (Eshtiagh-Hosseini, Necas *et al.*, 2010). There are some hydrogen bonding interactions such as O–H···O and N–H···O between cations, anions and uncoordinated water molecules (Table 2). The water molecules act also as bridging agents and link anions and cationic fragments together *via* hydrogen bonds which resulted in the creation of six supramolecular synthons as *R*^2^_2_(8), *R*^3^_4_(10), *R*^3^_5_(10), *R*^4^_5_(15), *R*^4^_4_(18), *R*^4^_4_(26) (Figs. 2, 3). As it is seen in Fig. 4, there are also π-π   stacking interactions between the aromatic rings of the coordinated (pyzdc)^2–^ and carboxylate functional group anions and (2a-6mpyH)^+^ cation. Ion pairing, hydrogen bonds, π–π stacking, and van der Waals interactions stabilize the  crystal structure. These interactions lead to formation of a three-dimensional structure. By the help of hydrogen bond interactions between uncoordinated water molecules, the related crystalline network bears (H_2_O)_6_ cluster in the form of two branched-cyclohexane (Eshtiagh-Hosseini, Gschwind *et al.*, 2010).

### Experimental

A solution of pyzdcH_2_ (0.6 mmol, 0.1 g) and 2a-6mpy (1.2 mmol, 0.13 g) in water (10 ml) was refluxed for an hour, then a solution of CoCl_2_.6H_2_O (0.02 mmol, 0.05 g) was added dropwise and continued refluxing for 6 hrs at 293 K. The obtained orange solution gave orange block like crystals of title compound after slow evaporation of solvent at R.T.

### Refinement

Carbon bound hydrogen atoms were positioned geometrically and refined as riding using standard *SHELXTL* constraints, with their *U*_iso_ set to either 1.2U_eq_(C) or 1.5U_eq_(C_methyl_) of their parent atoms. The C—H distances were set to 0.93 and 0.96Å for aromatic and methyl H atoms, respectively. Hydrogen atoms bonded to N and O were located in a difference Fourier map and refined isotropically.

### Crystal data

**Table:**

(C_6_H_9_N_2_)_2_[Co(C_6_H_2_N_2_O_4_)_2_(H_2_O)_2_]·8H_2_O	*Z* = 1
*M**_r_* = 789.58	*F*(000) = 413
Triclinic, *P*1	*D*_x_ = 1.518 Mg m^−^^3^
Hall symbol: -P 1	Cu - *K*α radiation, λ = 1.54184 Å
*a* = 6.8570 (4) Å	Cell parameters from 3181 reflections
*b* = 10.2348 (5) Å	θ = 3–71°
*c* = 13.6403 (10) Å	µ = 4.68 mm^−^^1^
α = 109.604 (4)°	*T* = 150 K
β = 90.424 (5)°	Chunk, brown
γ = 105.524 (4)°	0.20 × 0.18 × 0.14 mm
*V* = 863.89 (9) Å^3^	

### Data collection

**Table:**

Rigaku RAPID II diffractometer	3151 reflections with > 2.0σ(*I*)
confocal optics	*R*_int_ = 0.036
ω scans	θ_max_ = 71.9°, θ_min_ = 3.5°
Absorption correction: multi-scan (*SCALEPACK*; Otwinowski & Minor, 1997)	*h* = 0→8
*T*_min_ = 0.280, *T*_max_ = 0.508	*k* = −12→12
19137 measured reflections	*l* = −16→16
3152 independent reflections	

### Refinement

**Table:**

Refinement on *F*^2^	0 restraints
Least-squares matrix: full	H atoms treated by a mixture of independent and constrained refinement
*R*[*F*^2^ > 2σ(*F*^2^)] = 0.030	*w* = 1/[σ^2^(*F*_o_^2^) + (0.057*P*)^2^ + 0.2735*P*] where *P* = (*F*_o_^2^ + 2*F*_c_^2^)/3
*wR*(*F*^2^) = 0.087	(Δ/σ)_max_ = 0.001
*S* = 1.04	Δρ_max_ = 0.23 e Å^−^^3^
3152 reflections	Δρ_min_ = −0.40 e Å^−^^3^
285 parameters	

### Special details

**Table:**

Geometry. All e.s.d.'s (except the e.s.d. in the dihedral angle between two l.s. planes) are estimated using the full covariance matrix. The cell e.s.d.'s are taken into account individually in the estimation of e.s.d.'s in distances, angles and torsion angles; correlations between e.s.d.'s in cell parameters are only used when they are defined by crystal symmetry. An approximate (isotropic) treatment of cell e.s.d.'s is used for estimating e.s.d.'s involving l.s. planes.
Refinement. Refinement on *F*^2^ for ALL reflections except for 0 with very negative *F*^2^ or flagged by the user for potential systematic errors. Weighted *R*-factors *wR* and all goodnesses of fit *S* are based on *F*^2^, conventional *R*-factors *R* are based on *F*, with *F* set to zero for negative *F*^2^. The observed criterion of *F*^2^ > σ(*F*^2^) is used only for calculating R_factor_obs *etc*. and is not relevant to the choice of reflections for refinement. *R*-factors based on *F*^2^ are statistically about twice as large as those based on *F*, and *R*- factors based on ALL data will be even larger.

### Fractional atomic coordinates and isotropic or equivalent isotropic displacement parameters (Å^2^)

**Table:**

	*x*	*y*	*z*	*U*_iso_*/*U*_eq_	
Co1	1.0000	0.5000	0.0000	0.01861 (12)	
O1W	0.78751 (19)	0.34893 (13)	−0.12293 (10)	0.0281 (3)	
O21	0.79587 (17)	0.45953 (12)	0.10552 (10)	0.0244 (3)	
O22	0.70494 (18)	0.32395 (14)	0.20508 (10)	0.0309 (3)	
O2W	0.4756 (2)	0.56148 (13)	0.22356 (12)	0.0277 (3)	
O31	0.97825 (17)	0.20966 (12)	0.31826 (9)	0.0257 (3)	
O32	0.77764 (18)	0.02378 (12)	0.18707 (10)	0.0278 (3)	
O3W	0.6610 (2)	0.37101 (15)	0.42401 (11)	0.0318 (3)	
O4W	0.3166 (2)	0.44434 (16)	0.37295 (12)	0.0307 (3)	
O5W	0.3645 (2)	0.92255 (14)	0.12233 (11)	0.0301 (3)	
N1	1.08935 (19)	0.33770 (13)	0.03478 (10)	0.0185 (3)	
N4	1.1850 (2)	0.14113 (14)	0.11111 (11)	0.0212 (3)	
N11	0.82673 (19)	0.07875 (14)	0.46263 (11)	0.0201 (3)	
N12	0.6818 (2)	−0.13230 (16)	0.32292 (12)	0.0243 (3)	
C2	0.9819 (2)	0.29538 (15)	0.10633 (12)	0.0179 (3)	
C3	1.0303 (2)	0.19613 (16)	0.14422 (13)	0.0188 (3)	
C5	1.2920 (2)	0.18647 (17)	0.04140 (13)	0.0219 (3)	
C6	1.2436 (2)	0.28428 (17)	0.00249 (13)	0.0213 (3)	
C12	0.7163 (2)	−0.06269 (17)	0.42549 (13)	0.0200 (3)	
C13	0.6442 (2)	−0.12847 (18)	0.49910 (14)	0.0240 (4)	
C14	0.6901 (3)	−0.0497 (2)	0.60314 (15)	0.0286 (4)	
C15	0.8071 (3)	0.0960 (2)	0.63821 (14)	0.0281 (4)	
C16	0.8729 (2)	0.15970 (18)	0.56645 (14)	0.0239 (3)	
C17	0.9948 (3)	0.31426 (18)	0.59367 (16)	0.0307 (4)	
C21	0.8125 (2)	0.36383 (17)	0.14298 (13)	0.0215 (3)	
C31	0.9158 (2)	0.13968 (17)	0.22335 (13)	0.0211 (3)	
H5	1.4018	0.1516	0.0183	0.026*	
H6	1.3200	0.3128	−0.0467	0.026*	
H11	0.869 (3)	0.117 (2)	0.4202 (17)	0.023 (5)*	
H13	0.5660	−0.2248	0.4768	0.029*	
H14	0.6432	−0.0930	0.6518	0.034*	
H15	0.8394	0.1485	0.7095	0.034*	
H121	0.618 (3)	−0.223 (3)	0.3014 (17)	0.032 (5)*	
H122	0.716 (3)	−0.086 (3)	0.2827 (19)	0.035 (6)*	
H17A	1.1225	0.3185	0.5646	0.046*	
H17B	1.0194	0.3600	0.6684	0.046*	
H17C	0.9210	0.3636	0.5655	0.046*	
H1W1	0.712 (4)	0.374 (3)	−0.150 (2)	0.044 (7)*	
H1W2	0.729 (4)	0.263 (3)	−0.1249 (19)	0.045 (7)*	
H2W1	0.561 (4)	0.520 (3)	0.191 (2)	0.052 (7)*	
H2W2	0.431 (4)	0.528 (3)	0.260 (2)	0.037 (7)*	
H3W1	0.728 (4)	0.345 (3)	0.378 (2)	0.046 (7)*	
H3W2	0.564 (4)	0.389 (3)	0.399 (2)	0.050 (7)*	
H4W1	0.300 (4)	0.493 (3)	0.430 (2)	0.039 (7)*	
H4W2	0.219 (4)	0.375 (3)	0.356 (2)	0.051 (7)*	
H5W1	0.478 (4)	0.970 (3)	0.145 (2)	0.049 (7)*	
H5W2	0.304 (4)	0.977 (3)	0.125 (2)	0.042 (7)*	

### Atomic displacement parameters (Å^2^)

**Table:**

	*U*^11^	*U*^22^	*U*^33^	*U*^12^	*U*^13^	*U*^23^
Co1	0.02261 (19)	0.01812 (18)	0.0174 (2)	0.00806 (13)	0.00137 (13)	0.00745 (14)
O1W	0.0329 (6)	0.0208 (6)	0.0290 (8)	0.0054 (5)	−0.0089 (5)	0.0086 (5)
O21	0.0262 (6)	0.0268 (6)	0.0269 (7)	0.0142 (5)	0.0064 (5)	0.0126 (5)
O22	0.0295 (6)	0.0455 (7)	0.0302 (8)	0.0197 (5)	0.0137 (5)	0.0219 (6)
O2W	0.0279 (6)	0.0239 (6)	0.0322 (8)	0.0108 (5)	0.0006 (5)	0.0083 (6)
O31	0.0292 (6)	0.0268 (6)	0.0191 (7)	0.0024 (5)	−0.0001 (5)	0.0100 (5)
O32	0.0292 (6)	0.0258 (6)	0.0242 (7)	−0.0011 (5)	−0.0008 (5)	0.0105 (5)
O3W	0.0355 (7)	0.0367 (7)	0.0249 (8)	0.0124 (6)	0.0078 (6)	0.0113 (6)
O4W	0.0306 (7)	0.0281 (6)	0.0273 (9)	0.0037 (6)	0.0009 (5)	0.0058 (6)
O5W	0.0247 (6)	0.0248 (6)	0.0455 (9)	0.0092 (5)	0.0019 (5)	0.0166 (6)
N1	0.0216 (6)	0.0175 (6)	0.0158 (7)	0.0058 (5)	0.0009 (5)	0.0051 (5)
N4	0.0232 (6)	0.0203 (6)	0.0211 (8)	0.0084 (5)	0.0008 (5)	0.0069 (5)
N11	0.0193 (6)	0.0221 (6)	0.0213 (8)	0.0071 (5)	0.0034 (5)	0.0098 (6)
N12	0.0257 (7)	0.0217 (7)	0.0235 (9)	0.0014 (6)	0.0001 (6)	0.0098 (6)
C2	0.0194 (7)	0.0173 (7)	0.0152 (9)	0.0045 (5)	0.0001 (6)	0.0040 (6)
C3	0.0193 (7)	0.0178 (7)	0.0167 (9)	0.0035 (5)	−0.0015 (6)	0.0042 (6)
C5	0.0224 (7)	0.0232 (7)	0.0205 (9)	0.0101 (6)	0.0038 (6)	0.0053 (6)
C6	0.0233 (7)	0.0223 (7)	0.0192 (9)	0.0081 (6)	0.0061 (6)	0.0070 (6)
C12	0.0157 (7)	0.0239 (7)	0.0232 (10)	0.0081 (6)	0.0024 (6)	0.0099 (7)
C13	0.0206 (7)	0.0264 (8)	0.0288 (11)	0.0072 (6)	0.0056 (6)	0.0139 (7)
C14	0.0259 (8)	0.0404 (10)	0.0286 (11)	0.0145 (7)	0.0109 (7)	0.0194 (8)
C15	0.0276 (8)	0.0383 (9)	0.0201 (10)	0.0159 (7)	0.0057 (7)	0.0072 (8)
C16	0.0200 (7)	0.0268 (8)	0.0260 (10)	0.0123 (6)	0.0021 (6)	0.0062 (7)
C17	0.0326 (9)	0.0248 (8)	0.0312 (11)	0.0111 (7)	−0.0016 (7)	0.0031 (7)
C21	0.0211 (7)	0.0243 (8)	0.0196 (9)	0.0086 (6)	0.0009 (6)	0.0066 (7)
C31	0.0214 (7)	0.0220 (7)	0.0230 (10)	0.0079 (6)	0.0019 (6)	0.0104 (7)

### Geometric parameters (Å, °)

**Table:**

Co1—O21^i^	2.0790 (12)	N11—C12	1.357 (2)
Co1—O21	2.0790 (11)	N11—C16	1.363 (2)
Co1—O1W	2.0841 (12)	N11—H11	0.82 (2)
Co1—O1W^i^	2.0841 (12)	N12—C12	1.325 (2)
Co1—N1	2.1045 (12)	N12—H121	0.86 (2)
Co1—N1^i^	2.1045 (12)	N12—H122	0.84 (2)
O1W—H1W1	0.77 (3)	C2—C3	1.393 (2)
O1W—H1W2	0.86 (3)	C2—C21	1.516 (2)
O21—C21	1.2760 (19)	C3—C31	1.518 (2)
O22—C21	1.228 (2)	C5—C6	1.388 (2)
O2W—H2W1	0.85 (3)	C5—H5	0.9300
O2W—H2W2	0.72 (3)	C6—H6	0.9300
O31—C31	1.256 (2)	C12—C13	1.410 (2)
O32—C31	1.244 (2)	C13—C14	1.361 (3)
O3W—H3W1	0.80 (3)	C13—H13	0.9300
O3W—H3W2	0.84 (3)	C14—C15	1.404 (3)
O4W—H4W1	0.80 (3)	C14—H14	0.9300
O4W—H4W2	0.80 (3)	C15—C16	1.365 (3)
O5W—H5W1	0.79 (3)	C15—H15	0.9300
O5W—H5W2	0.77 (3)	C16—C17	1.494 (2)
N1—C6	1.329 (2)	C17—H17A	0.9600
N1—C2	1.344 (2)	C17—H17B	0.9600
N4—C5	1.335 (2)	C17—H17C	0.9600
N4—C3	1.342 (2)		
			
O21^i^—Co1—O21	180.00 (7)	N4—C3—C2	121.24 (14)
O21^i^—Co1—O1W	90.50 (5)	N4—C3—C31	114.49 (13)
O21—Co1—O1W	89.50 (5)	C2—C3—C31	124.27 (14)
O21^i^—Co1—O1W^i^	89.50 (5)	N4—C5—C6	121.69 (14)
O21—Co1—O1W^i^	90.50 (5)	N4—C5—H5	119.20
O1W—Co1—O1W^i^	180.00 (6)	C6—C5—H5	119.20
O21^i^—Co1—N1	100.88 (5)	N1—C6—C5	120.75 (14)
O21—Co1—N1	79.12 (5)	N1—C6—H6	119.60
O1W—Co1—N1	92.48 (5)	C5—C6—H6	119.60
O1W^i^—Co1—N1	87.52 (5)	N12—C12—N11	119.07 (15)
O21^i^—Co1—N1^i^	79.12 (5)	N12—C12—C13	123.23 (15)
O21—Co1—N1^i^	100.88 (5)	N11—C12—C13	117.70 (15)
O1W—Co1—N1^i^	87.52 (5)	C14—C13—C12	119.36 (15)
O1W^i^—Co1—N1^i^	92.48 (5)	C14—C13—H13	120.30
N1—Co1—N1^i^	180.00 (6)	C12—C13—H13	120.30
Co1—O1W—H1W1	119.7 (19)	C13—C14—C15	121.07 (16)
Co1—O1W—H1W2	122.8 (17)	C13—C14—H14	119.50
H1W1—O1W—H1W2	109 (2)	C15—C14—H14	119.50
C21—O21—Co1	116.29 (10)	C16—C15—C14	119.19 (17)
H2W1—O2W—H2W2	110 (3)	C16—C15—H15	120.40
H3W1—O3W—H3W2	108 (3)	C14—C15—H15	120.40
H4W1—O4W—H4W2	104 (3)	N11—C16—C15	118.88 (15)
H5W1—O5W—H5W2	106 (3)	N11—C16—C17	116.76 (16)
C6—N1—C2	118.50 (13)	C15—C16—C17	124.35 (17)
C6—N1—Co1	128.65 (11)	C16—C17—H17A	109.50
C2—N1—Co1	112.57 (10)	C16—C17—H17B	109.50
C5—N4—C3	117.44 (13)	H17A—C17—H17B	109.50
C12—N11—C16	123.77 (15)	C16—C17—H17C	109.50
C12—N11—H11	117.9 (15)	H17A—C17—H17C	109.50
C16—N11—H11	118.3 (15)	H17B—C17—H17C	109.50
C12—N12—H121	117.3 (15)	O22—C21—O21	125.85 (15)
C12—N12—H122	119.2 (16)	O22—C21—C2	118.38 (14)
H121—N12—H122	123 (2)	O21—C21—C2	115.77 (13)
N1—C2—C3	120.37 (14)	O32—C31—O31	126.69 (15)
N1—C2—C21	116.14 (13)	O32—C31—C3	116.34 (15)
C3—C2—C21	123.47 (14)	O31—C31—C3	116.80 (13)
			
O21^i^—Co1—O21—C21	113 (47)	C3—N4—C5—C6	−1.4 (2)
O1W—Co1—O21—C21	93.19 (12)	C2—N1—C6—C5	0.3 (2)
O1W^i^—Co1—O21—C21	−86.81 (12)	Co1—N1—C6—C5	173.81 (11)
N1—Co1—O21—C21	0.57 (11)	N4—C5—C6—N1	0.9 (2)
N1^i^—Co1—O21—C21	−179.43 (11)	C16—N11—C12—N12	−179.22 (14)
O21^i^—Co1—N1—C6	3.94 (14)	C16—N11—C12—C13	0.6 (2)
O21—Co1—N1—C6	−176.06 (14)	N12—C12—C13—C14	178.72 (15)
O1W—Co1—N1—C6	94.93 (14)	N11—C12—C13—C14	−1.1 (2)
O1W^i^—Co1—N1—C6	−85.07 (14)	C12—C13—C14—C15	0.3 (2)
N1^i^—Co1—N1—C6	85 (62)	C13—C14—C15—C16	1.0 (2)
O21^i^—Co1—N1—C2	177.76 (10)	C12—N11—C16—C15	0.7 (2)
O21—Co1—N1—C2	−2.24 (10)	C12—N11—C16—C17	−179.59 (14)
O1W—Co1—N1—C2	−91.26 (11)	C14—C15—C16—N11	−1.5 (2)
O1W^i^—Co1—N1—C2	88.74 (11)	C14—C15—C16—C17	178.83 (15)
N1^i^—Co1—N1—C2	−102 (62)	Co1—O21—C21—O22	−179.10 (14)
C6—N1—C2—C3	−0.9 (2)	Co1—O21—C21—C2	1.06 (18)
Co1—N1—C2—C3	−175.43 (11)	N1—C2—C21—O22	177.04 (14)
C6—N1—C2—C21	177.95 (13)	C3—C2—C21—O22	−4.1 (2)
Co1—N1—C2—C21	3.44 (16)	N1—C2—C21—O21	−3.1 (2)
C5—N4—C3—C2	0.7 (2)	C3—C2—C21—O21	175.73 (14)
C5—N4—C3—C31	−179.98 (13)	N4—C3—C31—O32	−85.16 (17)
N1—C2—C3—N4	0.4 (2)	C2—C3—C31—O32	94.11 (19)
C21—C2—C3—N4	−178.37 (14)	N4—C3—C31—O31	90.54 (17)
N1—C2—C3—C31	−178.80 (14)	C2—C3—C31—O31	−90.20 (19)
C21—C2—C3—C31	2.4 (2)		

Symmetry codes: (i) −*x*+2, −*y*+1, −*z*.

### Hydrogen-bond geometry (Å, °)

**Table:**

*D*—H···*A*	*D*—H	H···*A*	*D*···*A*	*D*—H···*A*
N11—H11···O31	0.82 (2)	1.98 (2)	2.794 (3)	179 (2)
N12—H121···O2W^ii^	0.86 (3)	2.05 (2)	2.900 (2)	170 (2)
N12—H122···O32	0.84 (3)	1.97 (3)	2.804 (3)	176 (2)
O1W—H1W1···O2W^iii^	0.78 (3)	1.99 (3)	2.770 (3)	178 (3)
O1W—H1W2···O5W^iii^	0.85 (3)	1.85 (3)	2.697 (2)	172 (3)
O2W—H2W1···O21	0.85 (3)	2.10 (3)	2.942 (3)	168 (3)
O2W—H2W2···O4W	0.72 (3)	2.07 (3)	2.784 (3)	176 (2)
O3W—H3W2···O4W	0.83 (3)	1.99 (3)	2.811 (3)	167 (3)
O4W—H4W1···O3W^iv^	0.80 (3)	1.98 (2)	2.755 (2)	165 (3)
O4W—H4W2···O31^v^	0.80 (2)	1.94 (2)	2.738 (2)	178 (3)
O5W—H5W1···O32^vi^	0.79 (2)	2.00 (3)	2.767 (2)	161 (3)
O5W—H5W2···N4^vii^	0.77 (3)	2.11 (3)	2.871 (3)	167 (3)

Symmetry codes: (ii) *x*, *y*−1, *z*; (iii) −*x*+1, −*y*+1, −*z*; (iv) −*x*+1, −*y*+1, −*z*+1; (v) *x*−1, *y*, *z*; (vi) *x*, *y*+1, *z*; (vii) *x*−1, *y*+1, *z*.
